# An interpretable clinical ultrasound-radiomics combined model for diagnosis of stage I cervical cancer

**DOI:** 10.3389/fonc.2024.1353780

**Published:** 2024-05-23

**Authors:** Xianyue Yang, Chuanfen Gao, Nian Sun, Xiachuan Qin, Xiaoling Liu, Chaoxue Zhang

**Affiliations:** ^1^ Department of Ultrasound, The First Affiliated Hospital of Anhui Medical University, Hefei, Anhui, China; ^2^ Department of Ultrasound, Anhui Provincial Maternity and Child Health Hospital, Hefei, Anhui, China; ^3^ Department of Ultrasound, Nanchong Central Hospital (Beijing Anzhen Hospital Nanchong Hospital), The Second Clinical Medical College, North Sichuan Medical College (University), Nanchong, Sichuan, China

**Keywords:** cervical cancer, ultrasound, machine learning, radiomics, Shapley additive explanations

## Abstract

**Objective:**

The purpose of this retrospective study was to establish a combined model based on ultrasound (US)-radiomics and clinical factors to predict patients with stage I cervical cancer (CC) before surgery.

**Materials and methods:**

A total of 209 CC patients who had cervical lesions found by transvaginal sonography (TVS) from the First Affiliated Hospital of Anhui Medical University were retrospectively reviewed, patients were divided into the training set (n = 146) and internal validation set (n = 63), and 52 CC patients from Anhui Provincial Maternity and Child Health Hospital and Nanchong Central Hospital were taken as the external validation set. The clinical independent predictors were selected by univariate and multivariate logistic regression analyses. US-radiomics features were extracted from US images. After selecting the most significant features by univariate analysis, Spearman’s correlation analysis, and the least absolute shrinkage and selection operator (LASSO) algorithm, six machine learning (ML) algorithms were used to build the radiomics model. Next, the ability of the clinical, US-radiomics, and clinical US-radiomics combined model was compared to diagnose stage I CC. Finally, the Shapley additive explanations (SHAP) method was used to explain the contribution of each feature.

**Results:**

Long diameter of the cervical lesion (L) and squamous cell carcinoma-associated antigen (SCCa) were independent clinical predictors of stage I CC. The eXtreme Gradient Boosting (Xgboost) model performed the best among the six ML radiomics models, with area under the curve (AUC) values in the training, internal validation, and external validation sets being 0.778, 0.751, and 0.751, respectively. In the final three models, the combined model based on clinical features and rad-score showed good discriminative power, with AUC values in the training, internal validation, and external validation sets being 0.837, 0.828, and 0.839, respectively. The decision curve analysis validated the clinical utility of the combined nomogram. The SHAP algorithm illustrates the contribution of each feature in the combined model.

**Conclusion:**

We established an interpretable combined model to predict stage I CC. This non-invasive prediction method may be used for the preoperative identification of patients with stage I CC.

## 1 Introduction

Cervical cancer (CC) is the fourth most common cancer in women worldwide, following breast, colorectal, and lung cancers (1), and is one of the most common gynecological malignancies (2, 3). Approximately 570,000 new cases of CC and 311,000 deaths occurred in 2018, and China is among the countries with the greatest burden of CC (4). Currently, the most common CC stage is based on the International Federation of Gynecology and Obstetrics (FIGO) 2018 system. It is divided into four stages, among which stage I refers to carcinoma strictly confined to the cervix uteri (extension to the corpus should be disregarded) (5), which belongs to early-stage CC, with a good prognosis and 5-year survival rate of approximately 90%, and it is only approximately 30% of them with lymph node metastasis (LNM) (6). Therefore, the early diagnosis of CC is particularly important. Ultrasound (US), computed tomography (CT), magnetic resonance imaging (MRI), and positron emission tomography/computed tomography (PET/CT) are all routinely used for CC examinations, but because the imaging pattern has inherent limitations, based on the traditional visual assessment, imaging technology cannot identify the grayscale images, which beyond the scope of human eye recognition, in disease diagnosis and treatment value is limited, hence the need more advanced tools to improve the existing imaging technology.

In recent years, radiomics has gradually become a research hotspot, which is a widely used and high-throughput method for medical imaging now (7). Using radiomics methods can extract quantitative imaging features based on intensity, shape, size volume texture features, etc. The feature redundancy, dimensionality reduction, pre-processing, and machine learning (ML)-based classification combining the extracted features can establish robust and clinically relevant models (8–10). In recent years, radiomics has been reported to achieve higher precision in the diagnosis, staging, and prognosis of many tumors (11–14). The standard ML consisting of artificial intelligence is a black-box prediction that is difficult to interpret by clinicians. Due to its ability to interpret and visualize the predictions of a model and to illustrate the contribution of each feature in the model, the Shapley additive explanations (SHAP) algorithm is currently the most recommended for model interpretation. The SHAP values can reveal the individual contribution of each feature to the model output of each observer (15, 16).

Studies have shown that radiomics features extracted from MRI or PET/CT images can be used to predict lymph node status (LNS) (17), tumor stage (18), histological subtype (19), and outcomes of neoadjuvant chemoradiation in patients with CC (20, 21). It has also been shown that the radiomics features extracted from US images can be used to predict preoperative LNS in early-stage CC patients (22). However, to the best of our knowledge, no studies have reported the use of US-radiomics and SHAP for explaining and visualizing the diagnosis of CC staging. Therefore, this study aims to build and validate an interpretable clinical US-radiomics combined model for personalized non-invasive assessment for the detection of stage I CC.

## 2 Materials and method

### 2.1 Patients

This study was approved by the Institutional Review Committee of the First Affiliated Hospital of Anhui Medical University (approval number PJ2023–07-11). The requirement for informed consent was waived because of the retrospective study design and use of known data. By searching the electronic medical records, CC patients who had cervical lesions found by transvaginal sonography (TVS) in the US Department of the First Affiliated Hospital of Anhui Medical University (Hospital 1) from June 2018 to September 2023 and CC patients of Anhui Provincial Maternity and Child Health Hospital (Hospital 2) and Nanchong Central Hospital (Hospital 3) from January 2018 to October 2023 were analyzed retrospectively.

**Figure 1:**
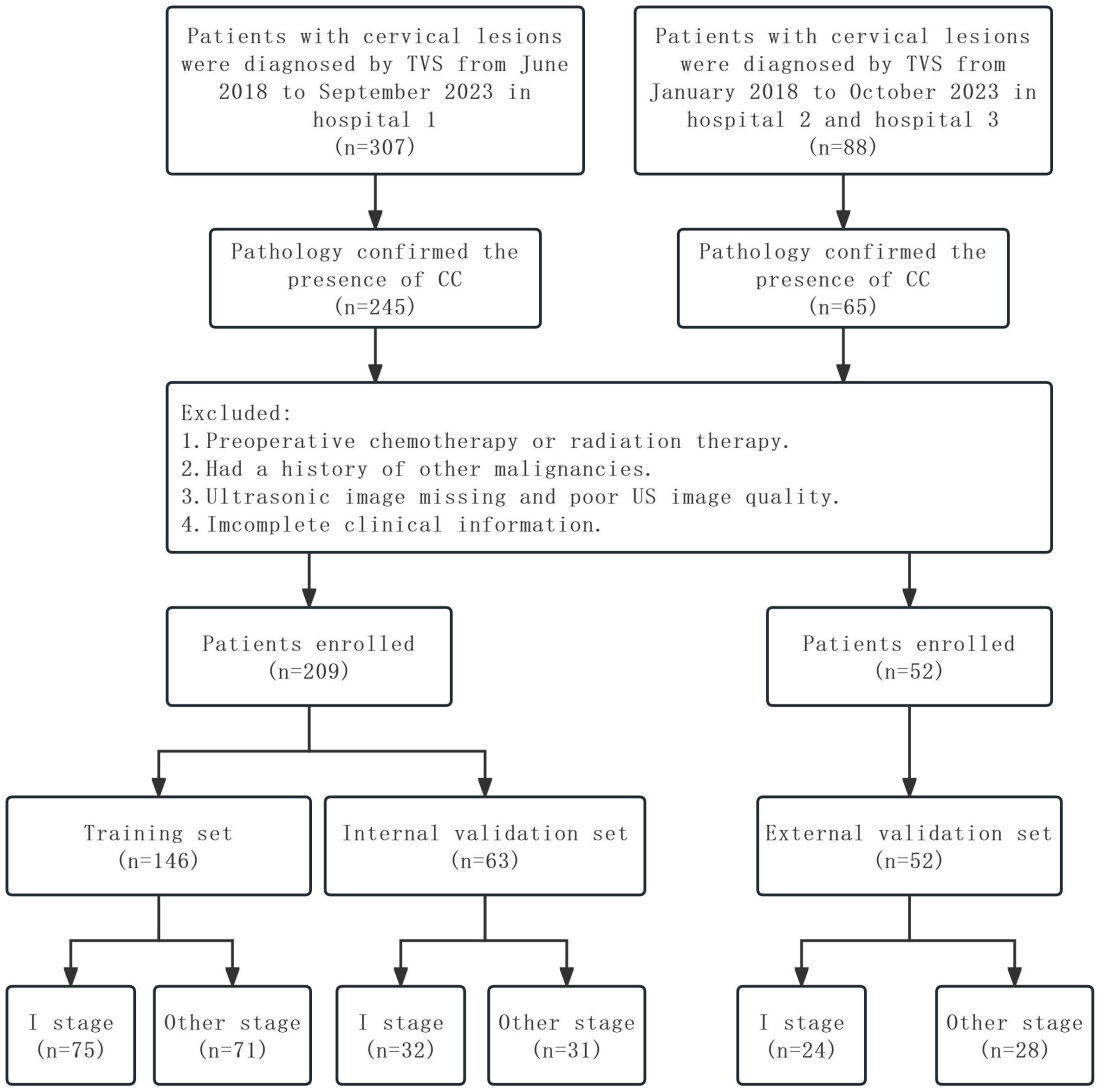
The flow diagram of the study.

The inclusion criteria for this study were as follows: 1) pathologically confirmed CC patients who underwent radical hysterectomy, 2) cervical biopsy-confirmed CC patients, and 3) TVS was performed less than 2 weeks before the hysterectomy or biopsy. The exclusion criteria were as follows: 1) chemotherapy or radiotherapy before US examination, 2) patients with a history of other malignancies, 3) the US images were lost or of poor quality, and 4) incomplete clinical information. Ultimately, 209 CC patients were enrolled from Hospital 1, and patients were randomly divided into the training and internal validation sets at a ratio of 7:3. Fifty-two CC patients were enrolled from Hospital 2 and Hospital 3 as the external validation set. The following baseline data were collected: age, SCCa, FIGO stage, histological type, and L by TVS. The study flowchart is shown in **Figure 1**.

### 2.2 Image acquisition

TVS was performed using a US probe with a frequency of 5–9 MHz. The patients’ bladder must be empty within 10 minutes before the examination; the lithotomy position was taken; a little coupling agent was placed on the top of the probe, a condom was placed, and a little coupling agent was placed on the top again; the probe was slowly inserted into the vagina until the vaginal vault was reached. The gynecological conditions in the machine were selected, and the appropriate depth was adjusted according to the size of the cervical lesion. First, the longitudinal section of the cervix was scanned, and then the transverse section was scanned. Careful and comprehensive observation of the cervical lesion was conducted, 6–10 clear and complete cervical lesion US images were collected for each patient, images were stored in a picture archiving and communication system (PACS), and one US image of the maximum section of the lesion was selected finally.

### 2.3 Image segmentation

**Figure 2:**
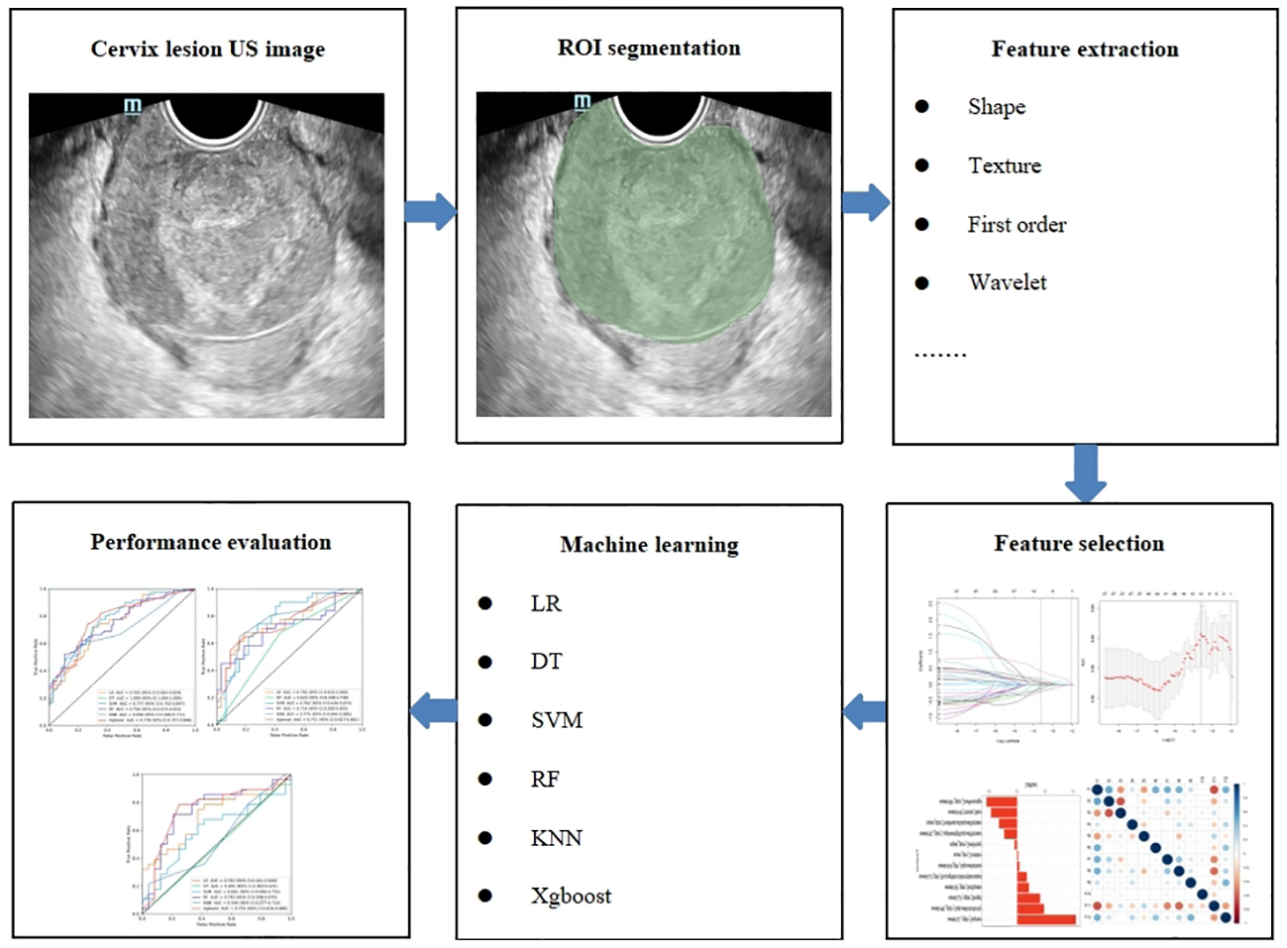
The radiomics flowchart of the study.

Cervical US image segmentation was completed by reader 1 (with 7 years of experience in gynecological US examination) and reader 2 (with 15 years of experience in gynecological US examination) using the 3D slicer 5.3.0 software. Regions of interest (ROIs) were manually selected and segmented (**Figure 2**). First, reader 1 and reader 2 delineated ROIs on the cervical US images of 50 randomly selected patients. Two weeks later, reader 1 again delineated the ROIs of these 50 patients. Intra- and inter-class correlation analyses were performed separately to test the consistency of extracted features between Reader 1 and Reader 2. The intra- and inter-class correlation coefficient (ICC) values were greater than 0.75, indicating a good consistency of the extracted features. The segmentation of the remaining cervical US images and the extraction of radiomics features were performed by reader 1 alone.

### 2.4 Radiomics feature extraction and selection

**Figure 3:**
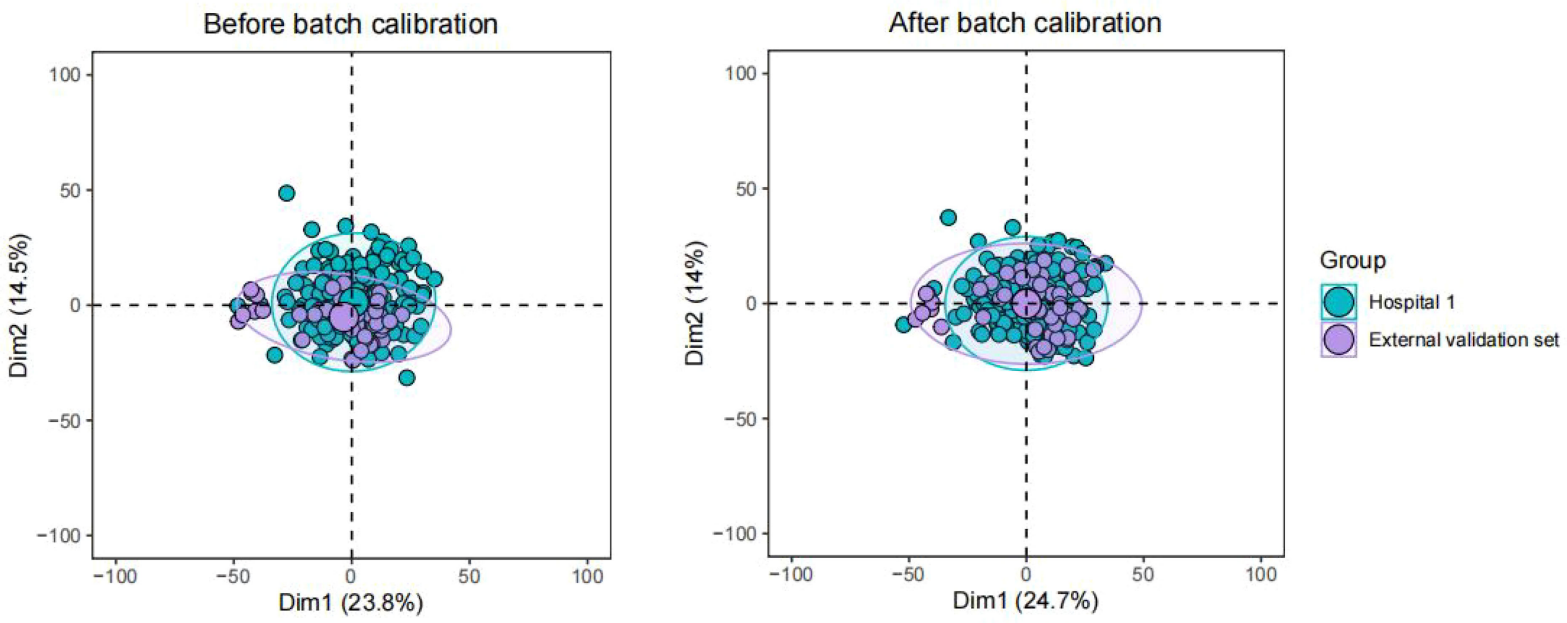
Batch effects were removed between Hospital 1 and the external validation set using Combat.

US-radiomics features were extracted using the Pyradiomics 3.0.1 software, which can extract a large number of features from the US images using many engineering algorithms (**Figure 2**). The images were standardized before feature extraction. First, features with ICC ≥ 0.75 were included in subsequent analyses. Second, batch effects were removed between Hospital 1 and the external validation set using Combat (**Figure 3**) (owing to very few cases originating from Hospital 3, the cases from Hospital 2 and Hospital 3 were consolidated as the external validation set). Then, univariate analysis was used for these features, with p < 0.05 features classified as significant correlation variables between stage I and non-stage I CC and included in the subsequent analyses. Again, Spearman’s correlation analysis was used to assess the correlation and redundancy of the features. If Spearman’s correlation coefficient ≥0.9, the variable was considered redundant and was excluded. Finally, the least absolute shrinkage and selection operator (LASSO) algorithm was applied for further feature selection (**Figure 2**).

### 2.5 Clinical model construction

First, univariate logistic regression analysis was used to analyze the correlation between clinical parameters and the CC stage. The variables with significant correlations (p < 0. 05) were included in the multivariate logistic regression analysis, and then independent predictors significantly associated with the CC stage were identified.

### 2.6 US-radiomics model construction

The selected features were trained using six ML algorithms, including linear regression (LR), decision tree (DT), support vector machine (SVM), random forest (RF), K-nearest neighbor (KNN), and eXtreme Gradient Boosting (Xgboost). Fivefold cross-validation (CV) was performed in the training data set to obtain the optimal parameter configuration. The hyperparameters of the six ML algorithms were adjusted using the grid search method and 10-fold CV. The patients in the testing data set were used to evaluate the performance of the model (**Figure 2**).

### 2.7 Statistical analysis

Statistical analyses were performed in the R 4.2.2 analysis platform. Quantitative data with a normal distribution were expressed as mean ± standard deviation, while quantitative data with a non-normal distribution were expressed as the median (interquartile range). Categorical data were expressed as numbers and percentages. For univariate analysis, the chi-square test, independent sample t-test, or the Mann–Whitney U test was used. Correlations were calculated using Spearman’s test. The DeLong test was used to compare the performance of the six ML algorithms and three models. The SHAP algorithm was run using the “Xgboost” and “SHAP” Python packages. For all tests, p < 0.05 was considered statically significant.

## 3 Results

### 3.1 Patients’ characteristics

According to the inclusion and exclusion criteria, in this study, 209 CC patients were enrolled from Hospital 1 and divided randomly into the training set (n = 146) and the internal validation set (n = 63). Fifty-two CC patients were enrolled from Hospital 2 and Hospital 3 as the external validation set. The detailed characteristics of these patients in the training, internal validation, and external validation sets are presented in **Table 1**. Patients with stage I CC in the training, internal validation, and external validation sets were 51.4%, 50.8%, and 46.2%, respectively.

**Table 1:** Detail characteristics in the training, internal validation, and external validation sets.

Set (N)		Training set (N = 146)			Internal validation set (N = 63)			External validation set (N = 52)		
Characteristic		C0 (N = 75)	C1 (N = 71)	p	C0 (N = 32)	C1 (N = 31)	p	C0 (N = 24)	C1 (N = 28)	p
Histological type (n, %)	Adenocarcinoma	10 (13.3%)	4 (5.6%)	0.177	7 (21.9%)	3 (9.7%)	1	3 (12.5%)	2 (7.1%)	1
	Adenosquamous carcinoma	1 (1.3%)	3 (4.2%)		0 (0%)	0 (0%)		0 (0%)	0 (0%)	
	Squamous cell carcinoma	64 (85.3%)	64 (90.1%)		25 (78.1%)	28 (90.3%)		21 (87.5%)	26 (92.9%)	
FIGO stage (n, %)	I	75 (100%)	0 (0%)	<0.001	32 (100%)	0 (0%)	1	24 (100%)	0 (0%)	1
	II	0 (0%)	33 (46.5%)		0 (0%)	19 (61.3%)		0 (0%)	19 (67.9%)	
	III	0 (0%)	32 (45.1%)		0 (0%)	9 (29%)		0 (0%)	5 (17.9%)	
	IV	0 (0%)	6 (8.5%)		0 (0%)	3 (9.7%)		0 (0%)	4 (14.3%)	
Age [median (IQR)]		53.00 (49.00–56.00)	55.00 (49.00–59.00)	0.209	53.00 (48.00– 61.75)	54.00 (48.00–58.00)	0.549	53.50 (46.00–57.75)	55.50 (46.75–66.50)	0.330
L [median (IQR)]		26.00 (20.50–31.00)	37.00 (26.50– 45.00)	<0.001	30.00 (23.00– 34.75)	39.00 (27.00–49.00)	<0.001	28.00 (25.00–37.75)	44.00 (34.25– 55.25)	0.003
SCCa [median (IQR)]		1.45 (0.95–2.73)	2.70 (1.05–7.45)	0.007	1.15 (0.64–2.10)	4.50 (1.35–9.65)	<0.001	2.75 (1.53– 4.30)	5.43 (2.40–9.59)	0.044
Rad-score [median (IQR)]		0.26 (0.21–0.33)	0.39 (0.32–0.51)	<0.001	0.29 (0.26–0.34)	0.41 (0.32–0.46)	<0.001	0.28 (0.23–0.35)	0.35 (0.35–0.41)	0.002

FIGO, International Federation of Gynecology and Obstetrics; IQR, interquartile range.

### 3.2 US-radiomics feature extraction and selection

A total of 944 radiomics features were extracted from US images. There were 920 features with ICC ≥ 0.75, then 131 related features were obtained through univariate analysis and removed by Spearman’s correlation analysis, and 38 imaging radiomics features were left. Finally, the following 12 features were identified as the most significant by the LASSO algorithm: wavelet.HHH_glszm_ZonePercentage, wavelet.HHH_firstorder_Mean, square_glszm_SmallAreaLowGrayLevelEmphasis, wavelet.HLL_glszm_LargeAreaHighGrayLevelEmphasis, original_glszm_ZoneEntropy, square_glcm_Correlation, wavelet.HLH_glrlm_GrayLevelVariance, wavelet.LLH_glrlm_RunLengthNonUniformityNormalized, wavelet.HLH_glcm_SumSquares, wavelet.LLH_ngtdm_Strength, wavelet.HHL_glszm_GrayLevelNonUniformity, and wavelet.LLL_ngtdm_Busyness. These were all included in the US-radiomics models.

### 3.3 Clinical model construction

In the univariate analysis, L (p < 0.001) and SCCa (p = 0.016) showed statistically significant differences between the C0 and C1 groups. The multivariate regression analysis showed that both L and SCCa were independent predictors of stage I CC.

### 3.4 Diagnostic performance of the US-radiomics models

The diagnostic performance of the six radiomics models based on different ML algorithms is shown in **Table 2**. The receiver operating characteristic (ROC) curves of these models in the training, internal validation, and external validation sets are shown in **Figure 2**. Among them, the Xgboost model performed the best, with area under the curve (AUC) values in the training, internal validation, and external validation sets of 0.778, 0.751, and 0.751, respectively.

**Table 2:** Performance of the six models in the training, internal validation, and external validation sets.

	Model	AUC (95% CI)	Accuracy	Sensitivity	Specificity
Training set	LR	0.745 (0.664–0.824)	0.712	0.732	0.693
	DT	1.000 (1.000–1.000)	1	1	1
	SVM	0.777 (0.702–0.847)	0.712	0.775	0.653
	RF	0.756 (0.679–0.831)	0.712	0.521	0.893
	KNN	0.694 (0.606–0.771)	0.685	0.549	0.813
	Xgboost	0.778 (0.707–0.849)	0.74	0.746	0.733
Internal validation set	LR	0.756 (0.632–0.869)	0.651	0.774	0.531
	DT	0.620 (0.509–0.748)	0.619	0.677	0.562
	SVM	0.762 (0.636–0.873)	0.73	0.903	0.562
	RF	0.716 (0.583–0.835)	0.683	0.484	0.875
	KNN	0.775 (0.664–0.885)	0.746	0.645	0.844
	Xgboost	0.751 (0.617–0.861)	0.619	0.71	0.531
External validation set	LR	0.702 (0.561–0.840)	0.635	0.571	0.708
	DT	0.491 (0.363–0.625)	0.5	0.607	0.375
	SVM	0.601 (0.446–0.755)	0.615	0.536	0.708
	RF	0.743 (0.598–0.870)	0.615	0.393	0.875
	KNN	0.544 (0.377–0.710)	0.538	0.25	0.875
	Xgboost	0.751 (0.610–0.880)	0.731	0.821	0.625

AUC, area under the curve; LR, linear regression; DT, decision tree; SVM, support vector machine; RF, random forest; KNN, K-nearest neighbor; Xgboost, eXtreme Gradient Boosting.

### 3.5 Clinical US-radiomics combined model

**Figure 4:**
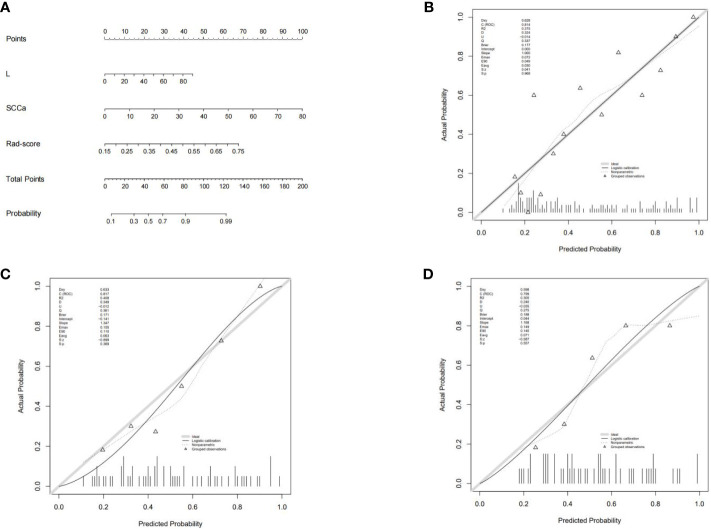
**(A)** The combined model nomogram. The values of clinical characteristics and rad-score can be converted into quantitative values according to the point axis. After summing the individual points to achieve the final sum shown on the total point axis, the evaluation of stage I CC is shown. **(B)** The calibration curve of the combined model in the training set. **(C)** The calibration curve of the combined model in the internal validation set. **(D)** The calibration curve of the combined model in the external validation set.

The combined model nomogram was established by combining the rad-score and clinical characteristics (**Figure 4A**). The calibration curve of the combined model all showed good agreement between the predicted and actual stage I CC in the three sets (**Figures 4B–D**).

### 3.6 Comparison of the three diagnostic models

**Figure 5:**
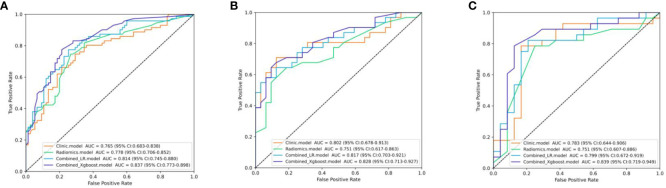
The ROC curves of the three models. **(A)** Three models’ ROC curves in the training set. **(B)** Three models’ ROC curves in the internal validation set. **(C)** Three models’ ROC curves in the external validation.

The differential validity of the three diagnostic models (clinical, US-radiomics, and combined) is shown in **Table 3**. The ROC curves of the three models in the training, internal validation, and external validation sets are shown in **Figure 5**. The combined model had the best diagnostic performance, with AUC values in the training, internal validation, and external validation sets of 0.837, 0.828, and 0.839, respectively.

**Table 3:** Performance of the clinical model, US-radiomics model, and combined model in the training, internal validation, and external validation sets.

	Model	AUC (95% CI)	Accuracy	Sensitivity	Specificity	p
Training set	Clinical model	0.765 (0.683–0.838)	0.726	0.746	0.707	0.001
	US-radiomics model	0.778 (0.706–0.852)	0.740	0.746	0.733	0.027
	Combined model	0.837 (0.773–0.898)	0.781	0.775	0.787	/
Internal validation set	Clinical model	0.802 (0.678–0.913)	0.683	0.806	0.562	0.417
	US-radiomics model	0.751 (0.617–0.863)	0.619	0.710	0.531	0.099
	Combined model	0.828 (0.713–0.927)	0.730	0.806	0.656	/
External validation set	Clinical model	0.783 (0.644–0.906)	0.750	0.929	0.542	0.130
	US-radiomics model	0.751 (0.607–0.886)	0.731	0.821	0.625	0.195
	Combined model	0.839 (0.719–0.949)	0.692	0.893	0.458	/

AUC, area under the curve.

### 3.7 Interpretation analysis and application of the Xgboost model

**Figure 6:**
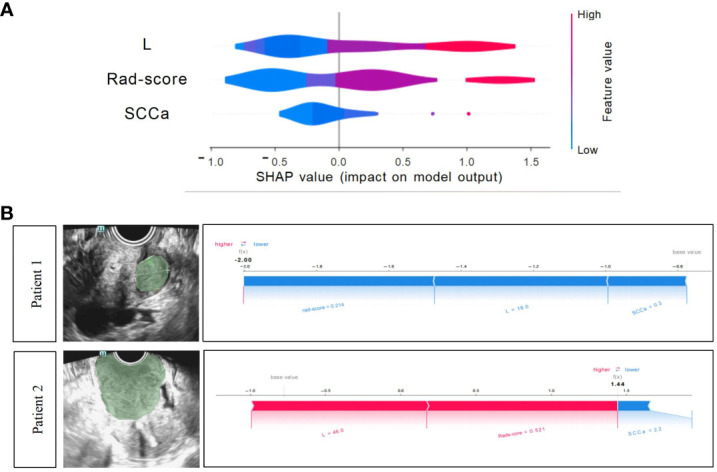
Xgboost model visualization and case application analysis. **(A)** Shapley summary diagram of the Xgboost combined model. A lower Shapley value (blue) suggests a greater tendency to stage I CC; conversely, a higher Shapley value (red) suggests a greater tendency to non-stage I CC. **(B)** Application analysis for two patients with CC of the Xgboost model.

The Shapley summary diagram in **Figure 6A** showed the contribution of three factors (L, rad-score, and SCCa) in predicting the CC stage for each case. The greater the absolute distribution range of Shapley values, the more important for the diagnosis of the CC stage. The SHAP force plot can explain the assessment of each case, and each feature is a force for the visualized Shapley value, which either increases (positive value) or decreases (negative value) the prediction from baseline. The baseline is the mean Shapley value for all the predicted features. The length of the arrow in **Figure 6B** indicates the contribution of a feature to the Shapley value, whereas the red and blue arrows indicate positive and negative, respectively (23).

### 3.8 Case application analysis of the Xgboost model

Two CC patients (**Figure 6B**), a 53-year-old woman with stage I CC and a 47-year-old woman with stage II CC, were selected for Xgboost model analysis. The cervical lesions of patient 1 had a short length (18 mm), low rad-score (0.214), and low SCCa (0.3 ng/mL), indicating a low SHAP value (−2.0), which strongly suggested that this patient was stage I CC and was consistent with the final pathological results. The cervical lesions of patient 2 had a long length (46 mm), high rad-score (0.521), and high SCCa (2.2 ng/mL), indicating a high SHAP value (1.44), which strongly suggested that this patient was non-stage I CC and was also consistent with the final pathological findings.

## 4 Discussion

It has been studied that MRI-based radiomics can diagnose early-stage CC. The accuracy of predicting the early stage of CC pre-operatively based on MRI-radiomics was 0.684 (18). The AUC values of T2-weighted images (T2WI)-based and apparent diffusion coefficient (ADC)-based radiomics model for predicting the early stage of CC were 0.855 and 0.823, respectively, in the training cohort and 0.861 and 0.81, respectively, in the cohort (24). In our study, the AUC values for predicting stage I CC in the training, internal validation, and external validation sets were 0.837, 0.828, and 0.839, respectively. These were generally consistent with the previous studies, but they did not set up an external validation group, and MRI is sometimes limited by cost or equipment availability. Ultrasonography is currently recognized as a convenient, quick, radiation-free, and affordable examination method to help clinicians detect and diagnose CC.

In this study, finally, we identified 12 radiomics features from cervical US images and used them to construct predictive models of six ML classification algorithms to detect stage I CC, in which the Xgboost model performed the best. Meanwhile, we included several clinical data, and the univariate and multivariate logistic regression analyses showed that L and SCCa were independent predictors in the clinical model. SCCa is a glycoprotein secreted by squamous cell carcinoma tissue. It plays an important role in the invasion, infiltration, and metastasis of squamous carcinoma cells. It is mainly used for the diagnosis of squamous cell carcinoma. There were only 24 cases of adenocarcinoma in this study, only 11%, and the histological type was not statistically significant between the C0 and CI groups. Therefore, the influence of histological type on the results of this study can be ignored. Previous studies have also shown that SCCa level was associated with CC progression and had some significance for the prediction of CC lymphatic metastasis (25), which is consistent with the results of this study.

We constructed an explainable Xgboost combined model incorporating important clinicoradiological factors and rad-score to predict stage I CC. The combined model improved the overall prediction effect of the model compared to the clinical and US-radiomics model, with AUC values as high as 0.837, 0.828, and 0.839 in the training, internal validation, and external validation sets, respectively. The personalized contribution of each feature was visualized using the Shapley algorithm for each case, which helps to explain the predictive power of the features in this model. Furthermore, the complex Xgboost combination model was visualized as a reliable clinical diagnostic support tool that we can easily apply.

Furthermore, we can boldly predict the specific stage of stage I CC based on the combined model established in this study and the size of the lesion so as to plan the need for lymph node dissection. Unnecessary lymph node dissection is associated with both short- and long-term complications, such as nerve injury, lower extremity lymphedema, and lymph sac formation (26, 27). Studies had shown that none of the 161 patients with preoperative MRI showing negative pelvic lymph node status, tumor size <20 mm, and squamous/adenosquamous histotype showed LNM (28).

Of course, there are also several limitations in our study. First, the retrospective analysis was prone to selection bias. Second, the number of samples was relatively small, and the different histological types were not studied separately. Third, radiomics has reproducibility problems. This study considered the reproducibility of extracted features, the US-radiomics features were extracted from ROIs, which were delineated by two senior ultrasonologists, the extracted features were evaluated by ICC, and ICC > 0.75 was considered reproducible. Meanwhile, the internal and external validation sets were set up, that is, considering the repeatability of the model. However, due to the retrospective analysis, the US images were not derived from the same machine, and the parameter settings were different, which also affected the reproducibility. This is our future research direction.

## 5 Conclusions

We used the Xgboost algorithm to construct an interpretable clinical US-radiomics combined model for predicting stage I CC and used the SHAP approach to quantify the contribution of each feature to the model. The satisfactory diagnostic performance of the model was also successfully validated on the independent external validation set. Therefore, our combined model is expected to assist clinicians in evaluating stage I CC non-invasively.

## Data availability statement

The original contributions presented in the study are included in the article/Supplementary Material. Further inquiries can be directed to the corresponding author.

## Ethics statement

The studies involving humans were approved by the Institutional Review Committee of the First Affiliated Hospital of Anhui Medical University. The studies were conducted in accordance with the local legislation and institutional requirements. Written informed consent for participation was not required from the participants or the participants’ legal guardians/next of kin in accordance with the national legislation and institutional requirements. Written informed consent was not obtained from the individual(s) for the publication of any potentially identifiable images or data included in this article because the requirement for informed consent was waived due to the retrospective study design and use of deidentified data.

## Author contributions

XY: Conceptualization, Formal Analysis, Investigation, Methodology, Software, Writing – original draft, Writing – review & editing. CG: Writing – review & editing, Formal Analysis, Software. NS: Writing – review & editing, Investigation. XQ: Investigation, Writing – review & editing, Methodology. XL: Methodology, Writing – review & editing, Formal Analysis, Software. CZ: Writing – review & editing, Conceptualization, Funding acquisition, Supervision.
